# Sex differences for antipsychotic dosing and how to guide therapy

**DOI:** 10.1192/j.eurpsy.2023.119

**Published:** 2023-07-19

**Authors:** G. Schoretsanitis

**Affiliations:** Department of Psychiatry, Psychotherapy and Psychosomatics, Psychiatric Hospital, University of Zurich, Zurich, Switzerland

## Abstract

**Abstract:**

Over the past decades the role of sex in the antipsychotic treatment type and dose selection has been virtually overseen. In fact, medication approval trials barely stratify for sex differences. Emerging hints from different types of data highlight the need for antipsychotic treatment personalization within the context of sex differences. Varying bioavaibility patterns of the prescribed antipsychotics between female and male patients may be easy to capture measuring plasma or serum levels of the antipsychotics ultimately guiding dose selection in clinical routine. Here we will discuss pitfalls and current evidence regarding sex affecting dose selection to enhance safety and effectiveness outcomes of antipsychotic treatment.

**Disclosure of Interest:**

G. Schoretsanitis Consultant of: HLS Therapeutics and ThermoFisher

